# Influence of Lifestyle on Brain Sensitivity to Circulating Insulin-like Growth Factor 1

**DOI:** 10.3390/ijms262010008

**Published:** 2025-10-14

**Authors:** Jonathan Zegarra-Valdivia, M. Zahid Khan, Aurora Putzolu, Raffaela Cipriani, Jaime Pignatelli, Ignacio Torres Aleman

**Affiliations:** 1 Achucarro Basque Center for Neuroscience, 48940 Leioa, Spain; zahid.khan@achucarro.org (M.Z.K.); aurora.putzolu@studenti.unimi.it (A.P.); raffaela.cipriani@achucarro.org (R.C.); 2 CIBERNED, Spain; jpigna@cajal.csic.es; 3 Psychology Department, Universidad Cientifica del Sur, Lima 15721, Peru; 4 Cajal Institute (CSIC), 28002 Madrid, Spain; 5 Ikerbasque Science Foundation, 48009 Bilbao, Spain

**Keywords:** insulin-like growth factor 1, lifestyle, brain, mood

## Abstract

Lifestyle factors, including social relationships and diet, influence mood homeostasis, a mechanism often dysregulated in high-incidence mental illnesses like depression and Alzheimer’s dementia (AD). Given that insulin-like growth factor 1 (IGF-1) modulates mood and its blood levels are altered in both AD and affective disorders, we investigated whether IGF-1 activity in the brain was affected in mice subjected to social isolation or a high-fat diet (HFD). We found that both lifestyle conditions increased anxiety and depression-like behavior in C57BL/6 mice of both sexes, as determined by the elevated zero maze/open field tests and the forced swim test, respectively. These lifestyle conditions were associated with loss of neuronal responses to systemic IGF-1. Enhanced neuronal activity in the prefrontal cortex—measured via Ca^++^ fiber photometry following intraperitoneal IGF-1 administration—was absent in both socially isolated and HFD-fed mice. However, only the HFD-fed group exhibited elevated serum IGF-1 levels. These findings suggest that loss of brain IGF-1 input may contribute to the observed mood disturbances, providing potential new targets to explore the heightened risk of depression and AD associated with loneliness and unhealthy diets in humans. Importantly, because reduced IGF-1 activity in the brain is not consistently reflected in serum levels, serum measurements are an unreliable indicator of brain IGF-1 activity.

## 1. Introduction

We previously proposed that IGF-1 signaling in the brain may contribute to the effects of lifestyle on brain health [1]. This hypothesis is supported by evidence that stress (linked to social intercourse) and inflammation (linked to diet) can impair IGF-1 activity [2]. Indeed, IGF-1 is a wide-spectrum neuromodulator, highly abundant in the circulation [3]. Circulating IGF-1 crosses the blood–brain barrier [4], and exerts important functions at central level, affecting energy balance [5], mood [6], cognition [7], and sociability [8]. Low serum IGF-1 levels increase vulnerability to stress [9], and cause depression [10], whereas obese [11] or depressed [12] individuals show either high bioactive IGF-1 or high serum IGF-1 levels, respectively, hinting to a status of IGF-1 resistance [13]. Recently, we found in mice that both stress [2], and overweight [2] markedly reduce the entrance of IGF-1 into the brain, pointing to an underlying brain IGF-1 resistance, probably at the blood–brain barrier (BBB).

We now investigated the effects of social isolation and a high-fat diet (HFD) on mood in adult mice of both sexes. We also examined how systemic IGF-1 influences brain function, as measured by neuronal activity, given the broad modulatory role of IGF-1 in this process. Both conditions altered mood and were associated with blunted neuronal activation in response to systemic IGF-1. However, only HFD-fed mice exhibited elevated serum IGF-1 levels. These findings highlight the importance of developing functional assays to assess site and tissue-specific IGF-1 activity—particularly in the brain—to elucidate its role in health and disease.

## 2. Results

### 2.1. Lifestyle Modulates Mood

**Figure 1:**
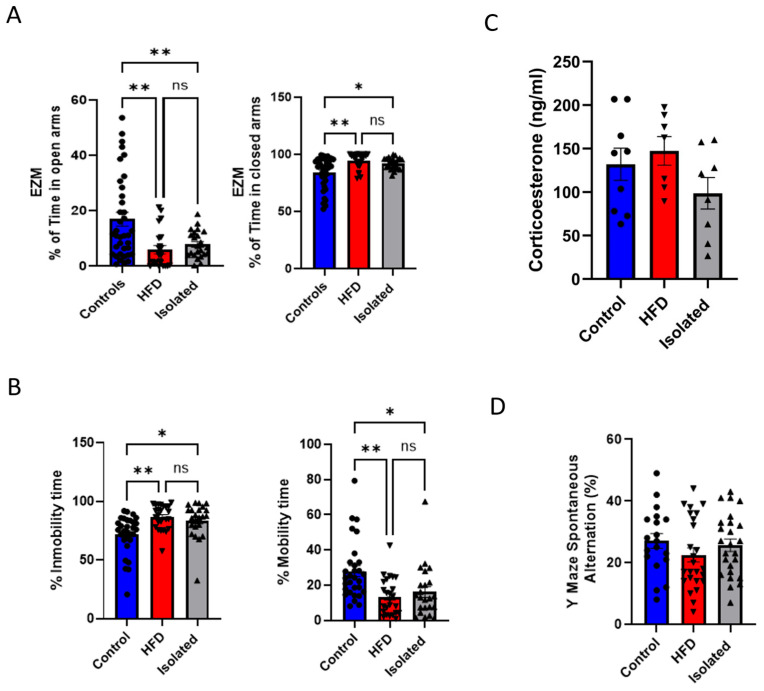
Effects of lifestyle on mood. (**A**) Anxiety levels in the different groups were measured in the Zero maze. Both isolated and HFD-fed mice showed higher anxiety, as determined by less time spent in the open arms of the maze (left histograms) and more in the closed arms (right histograms). Controls (n = 37), HFD (n = 22), Isolated (n = 23). One-way ANOVA showed significant group differences in time spent in open arms (F(2,79) = 8.54, *p* = 0.0004) and closed arms (F(2,78) = 7.87, *p* = 0.0008). Tukey’s post hoc test revealed that controls differed from both HFD and isolated groups (all *p* < 0.012), while HFD and isolated groups did not differ (*p* > 0.80). (**B**) Depression-like behavior was determined using the Forced Swim Test (FST). Both isolated and HFD-fed mice show increased depressive symptoms, as determined by less time struggling and swimming (right histograms) and more time immobile (left histograms), as compared to controls. Controls (n = 30), HFD (n = 25), Isolated (n = 23). One-way ANOVA revealed significant differences in immobility (F(2,75) = 8.10, *p* = 0.0007) and mobility (F(2,75) = 8.10, *p* = 0.0007). Tukey’s post hoc test indicated that both HFD and isolated groups differed significantly from controls (all *p* ≤ 0.018), while HFD and isolated animals did not differ (*p* > 0.78). (**C**) Serum corticosterone levels in the 3 experimental groups. Controls (n = 9), HFD (n = 7), Isolated (n = 8). One-way ANOVA did not show significant group differences (F(2,21) = 1.84, *p* = 0.184), and post hoc Tukey’s test confirmed that corticosterone levels were not significantly different among groups (all *p* > 0.17). (**D**) Spatial memory was assessed by measuring alternation behavior in the Y-maze test. No significant differences in alternation behavior were observed among groups compared to controls: Controls (n = 19), HFD (n = 25), Isolated (n = 25). One-way ANOVA confirmed the absence of group effects (F(2,66) = 1.05, *p* = 0.355), and Tukey’s post hoc test showed no significant pairwise differences (all *p* > 0.35), ns = not significant, * *p* < 0.05 and ** *p* < 0.01.

Mice fed a HFD began developing overweight phenotypes within 3–4 weeks (Figure S1A,B). As previously published by us [14,15], after approximately 10 weeks, HFD-fed mice will exhibit glucose intolerance accompanied by hyperinsulinemia and insulin resistance. A sexual dimorphism in food consumption was observed, (Figure S1C), and, as expected, females gain less weight than males (Figure S1B), suggesting that their energy expenditure is higher. However, since mood changes induced by isolation or HFD were independent of sex (Figure S2A,B), we combined data from males and females. Both conditions increased anxiety-like behavior in the Zero maze (Figure 1A), with a similar trend observed in the open field test (Figure S2C). In the forced swim test (FST), both experimental groups showed increased immobility time, indicative of depressive-like behavior (Figure 1B). Serum corticosterone levels did not differ significantly between males and females (Figure S3), nor between control and experimental groups (Figure 1C), indicating that the interventions did not induce systemic stress responses detectable through the basal activity of the HPA axis. Cognitive function was assessed using the Y-maze spontaneous alternation test (a measure of working memory), with no differences observed between experimental groups and controls (Figure 1D). No further tests of memory or learning were conducted.

### 2.2. Lifestyle Modulates Neuronal Responses to Systemic IGF-1

**Figure 2:**
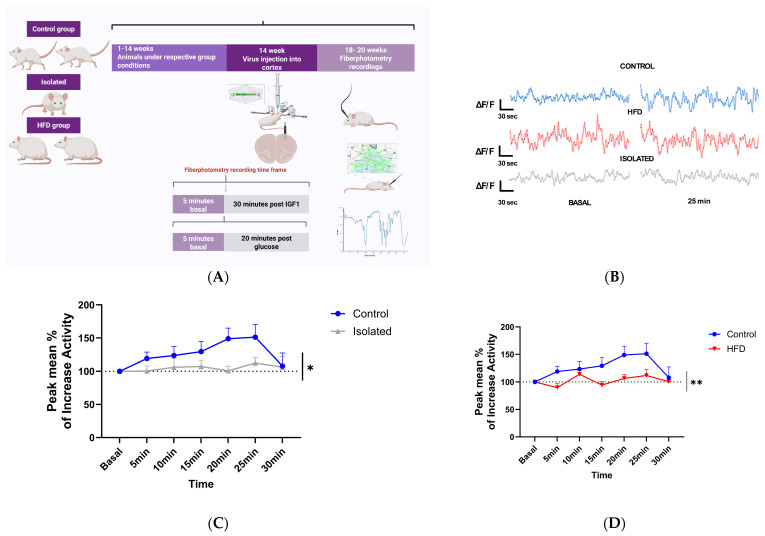
Neuronal responses to systemic IGF-1. (**A**) Diagram of fiber-photometry experimental set up. (**B**) Representative recordings of the three groups are shown at basal conditions and at 25 min. We chose the 25 min post-injection interval as previous reports indicate that systemic IGF-1 reliably enters the brain and modulates cortical and hypothalamic activity within 20–30 min [4,22,23]. This window also provided the most consistent neural responses in our pilot experiments. (**C**) Neuronal activity after i.p. IGF-1 administration was significantly increased in control mice compared to isolated mice. Two-way ANOVA revealed a significant main effect of group (F(1,96) = 6.81, *p* = 0.011), with no significant effects of time (F(6,96) = 1.07, *p* = 0.387) or interaction (F(6,96) = 0.70, *p* = 0.650). Post hoc Tukey’s test confirmed that control mice displayed a significantly greater increase in neuronal activity than isolated mice at 20 min after IGF-1 administration (*p* = 0.017). (**D**) HFD-fed mice did not respond to i.p. IGF-1 compared to controls; Controls (n = 11), HFD (n = 6), Isolated (n = 6). Two-way ANOVA revealed a significant main effect of group (F(1,100) = 9.79, *p* = 0.0023), with no significant effects of time or interaction. Complementary mixed-effects analysis, however, indicated that control mice exhibited significantly greater neuronal activation than HFD-fed mice at 5 and 20 min after IGF-1 administration (*p* = 0.028 and *p* = 0.032, respectively). * *p* < 0.05 and ** *p* < 0.01.

**Figure 3:**
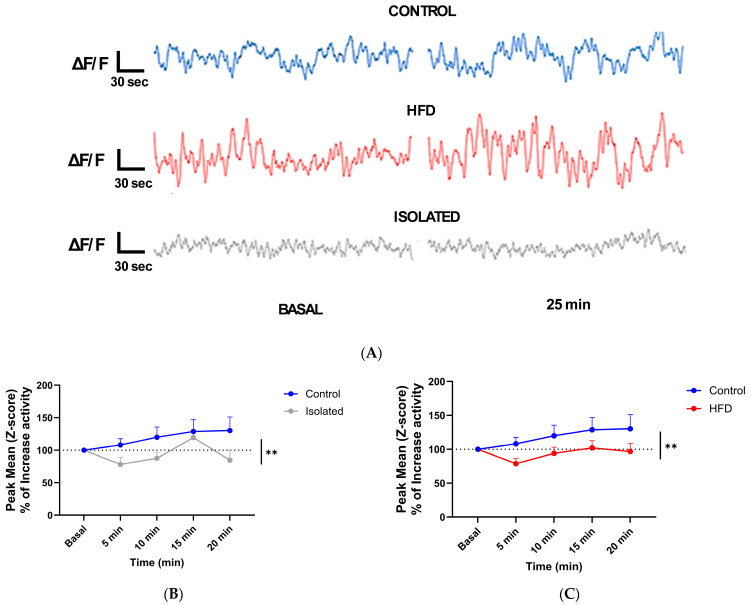
Neuronal responses to systemic Glucose. (**A**) Representative fiber-photometry traces over time are displayed, showing baseline activity and recordings taken at the 15 min mark. (**B**) Neuronal activity after i.p. glucose administration was significantly increased in control mice compared to isolated mice. Two-way ANOVA revealed a significant main effect of group (F(1,62) = 7.87, *p* = 0.0067). (**C**) HFD-fed mice did not respond to glucose load compared to control mice; Controls (n = 9), HFD (n = 9), Isolated (n = 7). Two-way ANOVA revealed a significant main effect of group (F(1,73) = 9.74, *p* = 0.0026); ** *p* < 0.01.

We next employed fiber photometry with virally transduced Ca^++^ indicators to monitor IGF-1’s effects on neuronal activity in the prefrontal cortex (PFC) across experimental groups. Calcium dynamics were measured as a proxy for neuronal firing patterns [16]. The PFC was chosen because is an area involved in mood homeostasis [17], influenced by social stress [18], and by diet [19]. Ca^++^ dynamics were monitored to determine PFC responses to a systemic (ip) bolus injection of IGF-1 (Figure 2A). While control mice showed neuronal activation following intraperitoneal IGF-1 administration, this response was abolished in both socially isolated and HFD-fed mice (Figure 2B,C). Furthermore, since IGF-1 is involved in brain glucose handling [20], and glucose stimulates neuronal activity [21], we tested whether glucose modulates neuronal activity in isolated or HFD-fed mice following the same procedure (Figure 2A), and found loss of responses to glucose too (Figure 3), which agrees with blunted brain IGF-1 activity.

**Figure 4:**
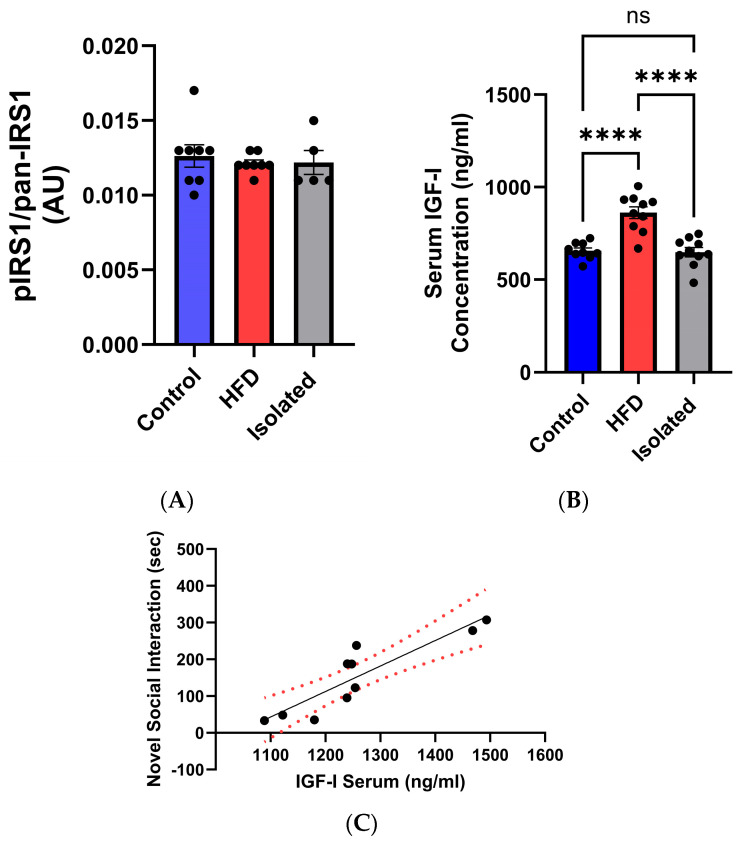
Modulation of IGF-1 activity by isolation and HFD. (**A**) pSer318IRS-1 levels in the cortex of the different experimental groups. Results are shown as the ratio of pSer318IRS1/total IRS1; Controls (n = 8), HFD (n = 8), Isolated (n = 5). Statistical analysis with the Kruskal–Wallis test did not detect significant group differences (H(2) = 0.35, *p* = 0.816), and post hoc Dunn’s tests confirmed the absence of significant pairwise differences (all *p* > 0.99). (**B**) Blood IGF-1 levels increased significantly in HFD-fed mice, but not in isolated mice; Controls (n = 9), HFD (n = 10), Isolated (n = 10). One-way ANOVA confirmed overall group differences (F(2,26) = 23.18, *p* < 0.0001, R^2^ = 0.64). Post hoc Tukey tests revealed that HFD-fed mice exhibited significantly higher IGF-1 levels compared to both controls (*p* < 0.0001) and isolated animals (*p* < 0.0001). In contrast, no significant difference was observed between controls and isolated mice (*p* = 0.979). (**C**) Serum IGF-1 levels significantly correlate in control mice with the level of social interaction in the social novelty test (n = 10). Linear regression analysis showed a significant positive correlation (F(1,8) = 31.56, *p* = 0.0005, R^2^ = 0.798), with higher serum IGF-1 levels predicting greater sociability; **** *p* < 0.0001.

To evaluate IGF-1 signaling, we quantified phosphorylation of IRS-1 at serine residues (pSer-IRS-1). As a key docking protein of the IGF-1 receptor, IRS-1 impairs IGF-1 signaling when serine-phosphorylated [24,25]. Cortical pSer^318^-IRS-1 levels were comparable across all experimental groups (Figure 4A). Consistent with previous reports [14], HFD-fed mice exhibited elevated serum IGF-1 levels, whereas socially isolated mice showed no significant changes in circulating IGF-1 (Figure 4B). The latter does not align with the observation that serum IGF-1 levels in control mice correlate with sociability (Figure 4C), as recently seen in humans [26].

## 3. Discussion

While lifestyle factors are known to influence brain health, their mechanistic underpinnings remain unclear. Our findings reveal that adverse lifestyle conditions—including social isolation and dietary imbalance—impair central IGF-1 signaling and are associated with affective disorders. As circulating IGF-1 access the brain in an activity-dependent manner [4,27], and modulates mood, cognition [7,9], and glucose handling [20], it seems likely that reduced brain sensitivity to circulating IGF-1 input contributes to affective disturbances that are commonly seen in stressed or overweight individuals [28,29], and that we also observed in this mouse study. Of note, when both detrimental lifestyle conditions are combined, a situation that is frequent in humans, the observed mood disturbances were the same as when mice were submitted to just one condition. 

Previous evidence documents dysregulated serum IGF-1 levels in both obese and depressed individuals [6,30,31]. While various mechanisms such as inflammation [32] and cellular senescence [33] have been proposed to connect obesity with mental illness, and diverse pathways (neuroinflammatory, neuroendocrine, epigenetic, and metabolic) link stress to psychiatric disorders [34], we propose that impaired central IGF-1 signaling represents an additional critical factor. This is supported by the known roles of IGF-1 in anti-inflammatory, antioxidant, and metabolic processes [35]. Notably, we observed blunted neuronal responses to systemic glucose in isolated and HFD-fed mice, suggesting compromised brain IGF-1 activity, consistent with its established role in mediating central glucose responses [20]. Since IGF-1 activity has been implicated in depression [6], AD [36] and other brain conditions [3], disruptions in brain activity of this growth factor may explain why loneliness and obesity are risk factors for these different psychiatric disorders [35].

We previously discussed that defining IGF-1 activity in the brain is not easy, although it could be a useful biomarker of brain health [35]. For instance, in previous studies in overweight mice, we observed high IGF-1 levels in serum, in parallel with normal levels in the brain. At the same time, these mice showed reduced brain IGF-1 activity -measured by receptor activation in response to systemic IGF-1 [14]. The present results corroborate these observations, and further illustrate the shortcomings of defining brain IGF-1 activity based solely on IGF-1 levels [37]. Using Ca^++^ fiber-photometry to determine neuronal responses to systemic IGF-1 we confirm that there is a loss of brain sensitivity to IGF-1 input in overweight mice and extend this observation to isolated mice. However, only overweight, but not isolated mice, show elevated serum IGF-1. The latter may apparently contrast with our previous observation that mice with low serum IGF-1 levels show reduced sociability [8], or that serum IGF-1 levels directly correlate with sociability (present observations), a trait recently documented in humans too [26]. Thus, while serum IGF-1 may affect sociability, social activity per se does not seem to influence serum IGF-1 levels.

The unaltered cortical pSer-IRS-1 levels in isolated and HFD-fed mice suggest that their impaired neuronal responses to systemic IGF-1 are not attributable to reduced IRS-1 tyrosine phosphorylation and consequent downstream signaling attenuation [38]. An alternative possibility is that reduced brain activity involves impaired transport of circulating IGF-1 across the blood–brain barrier (BBB) [4,39], analogous to the reported deficit in insulin CNS uptake [40], as systemic IGF-1 reaches the brain through this interface and constitutes a main IGF-1 gate of entrance to the brain. Given the well-established neuroprotective functions of circulating IGF-1 [41], compromised BBB transport may represent a fundamental pathophysiological mechanism common to various brain disorders, as we previously proposed [42]. However, more work is needed to firmly establish this possibility since reduced neuronal responsiveness to systemic IGF-1 could stem from other mechanisms, such as changes in receptor availability or sensitivity at other levels, or a combination thereof. We therefore propose impaired IGF-1 transport across the BBB as one possible explanation among several.

It has been shown that serum IGF-1 levels are increased in neuronal IGF-1R knock-out mice showing reduced responses to IGF-1 [43], suggesting that loss of brain IGF-1 activity is reflected on peripheral IGF-1 levels. Differential effects of HFD and isolation on serum IGF-1 levels, despite both showing loss of brain IGF-1 activity, may be explained by the fact that while obesity is reported to elicit IGF-1 resistance in the whole body [2], isolation stress may affect only brain sensitivity to IGF-1, limiting its systemic impact. Alternatively, changes in serum IGF-1 during social stress due to unwanted isolation (loneliness) in mice may require longer than 3 months of exposure to eventually impinge on systemic IGF-1 levels too. Indeed, isolated mice did not show elevated serum corticosterone, suggesting that they were not stressed. We previously reported loss of IGF-1R activation in response to systemic IGF-1 in mice submitted to predator exposure [2], while other authors found that combination of a more aggressive social stress (social intrusion) in obese mice induces stronger behavioral changes [44], which further suggests that 3 months of isolation stress is a comparatively mild disturbance.

Several limitations of our study should be noted. First, we did not directly assess glucose tolerance or insulin sensitivity in the current cohorts. These phenotypes are well established in HFD-fed mice [14], and insulin resistance is known to impact brain function and behavior through pathways overlapping with IGF-1 [45,46,47]. Therefore, the contribution of these alterations to brain responses to systemic IGF-1 should be explored. Indeed, impaired glucose homeostasis is a well-documented feature of both social isolation and HFD models. These established metabolic alterations form an important context for the interpretation of our findings on IGF-1 brain responsiveness. In addition, we did not measure blood insulin or IGF-1 dynamically after injection of glucose or IGF-1 since serial sampling would have interfered with fiber photometry recordings. Future studies combining minimally invasive blood collection with photometry would be valuable to disentangle systemic versus neuronal effects. Another limitation is that we did not measure IGF-1 levels in brain tissue. Future work combining direct measurement of brain IGF-1 with functional assessments such as these ones, will offer a more complete picture of IGF-1 dynamics in the brain. Moreover, the extended fasting protocol used for fiber-photometry recordings, while necessary to elicit robust neuronal responses in photometry, may itself act as a metabolic stressor [48]. This limitation should be considered when interpreting the results. Also, we only used the Y maze to test cognition (working memory) in this study because, in our hands, isolation or HFD did not alter this trait. Since previous results showed altered Y maze performance in isolated [49] or HFD-fed mice [50], and other cognitive traits also seem to be altered in these conditions [51,52], a more in-depth analysis of the cognitive status of these mice would help determine whether under our experimental conditions isolation and/or HFD alter cognition, as the Y maze test may lack sufficient sensitivity.

In summary, our results show reduced brain sensitivity to peripheral IGF-1 input in isolated and high-fat diet-fed mice, underscoring the need to develop reliable functional assays of IGF-1 activity in target organs such as the brain. This is important if we want to gain insight into the significance of this neurotrophic factor in brain health as a balance between peripheral and central IGF-1 activity has been postulated to be a key feature of its actions [53].

## 4. Materials and Methods

### 4.1. Animals

Adult male and female C57BL/6J mice (Janvier Labs, Paris, France) were used in a 1/1 ratio. Mice were kept in a room with controlled temperature (22 °C) under a 12–12 h light-dark cycle, and water ad libitum. Mice were fed with a standard lab diet or high fat diet (HFD) containing 45% fat in the diet + 1.25% cholesterol (ref E15744-34, sniff Spezialdiäten GmbH; Soest, Germany). All experimental protocols were performed during the light cycle between 12.00 and 17.00 h and reported in accordance with ARRIVE guidelines. All methods were carried out in accordance with relevant regulations (2010/63/EU) and were approved by the University of the Basque Country Bioethics Committee (M20-2022-009 PIBA). Sample size was determined by power calculations while minimizing animal numbers, where possible. Final group sizes reflect these considerations along with incidental loss of samples (see details below). Animals were not randomized due to the constraints associated with the experimental design (predefined lifestyle conditions, sequential measurements). Potential confounders such as litter, cage effects, order of handling, estrous cycle in females, subclinical infections, prenatal environment, ambient perturbations (noise, odors…etc.), and other subtle differences were not accounted for, and are acknowledged as limitations. Each trained experimenter took account of group allocation under study. All efforts were performed to reduce harm to the animals, and their health status was inspected daily. Mice were handled for three days prior to any experimental manipulations and familiarized with behavioral arenas to minimize novelty stress or deeply anesthetized with pentobarbital prior to sacrifice, when needed. Sample sizes were kept as little as possible to comply with current animal reduction policies. Although all efforts to alleviate suffering were implemented (expert handling and inspection, end-point deep anesthesia if needed), no adverse events were expected, nor found.

### 4.2. Experimental Design

No protocol of this study was prepared in advance. Experimental models used in this study aimed to mimic human lifestyle conditions of the general population in order to determine their relationship with brain IGF-1 activity. A control group was included in each set of experiments consisting of standard housing conditions, as described above. Mice were submitted to isolated (1 animal/cage) or standard group housing conditions (4–5/cage) and fed with standard or HFD diet for 16 weeks (Figure S4C). Various tests were used to determine mood traits (anxiety/depression)*,* and cognition. At the end of the experimental period, animals were deeply anesthetized (pentobarbital 50 mg/kg) in the morning, and blood and brain tissue were collected. End-point measures included checking reflexes in deeply anesthetized animals prior to culling. Mice remained under their respective housing (grouped or isolated) and diet (standard or HFD) conditions for the entire experimental period, including behavioral testing and final measurements (18–20 weeks). Serum corticosterone/IGF-1 and brain phosphor-Ser^318^IRS-1/IRS-1 were measured by ELISA (see below). While a total of 40 animals/group were used (20 females/20 males), not all the animals underwent all the procedures indicated in the experimental design figure, with varying sample sizes depending on the experiment. This was due to animal availability according to local animal house rules, the need to run varied procedures and measurements, and power analysis. Behavioral testing and fiber photometry were performed in partially overlapping cohorts; full allocation is provided in Table S1. No mortality occurred during the experimental period, including after viral transduction.

### 4.3. Behavioral Tests

Both males and females were analyzed together as no sex differences in the tested behaviors were appreciated. Group size of the experiments is indicated in the respective figure legends. After each trial, apparatuses were cleaned with 70% ethanol to remove scent cues. Mice were acclimated to the testing room for 30 min before the test, which was conducted under dim lighting.

*Zero maze*: To assess anxiety-like behavior in mice we evaluated their preference for open versus closed sections of an elevated, circular track. The maze consists of two open and two closed quadrants, elevated 40–60 cm above the floor, with each quadrant spanning 90°. Each mouse was placed at the border between an open and a closed section, facing the open area, and allowed to explore for 5 min while being video recorded. Time spent in open and closed sections was determined, calculating the percentage of time spent in open sections and the number of entries, with increased time in open sections indicating lower anxiety-like behavior and decreased time suggesting higher anxiety.

*Open field test*: Exploratory behavior and locomotion were assessed by introducing the animal to an open field arena (42 cm × 42 cm × 30 cm, Versamax; AccuScan Instruments, Inc., Columbus, OH, USA) for 10 min. All parameters were quantified as described [8]. Time spent exploring specific areas of the arena was measured.

*Forced swim test*: To assess depressive-like behavior, we used the forced swim test as described before [54]. Mice were placed in a glass cylinder (12 cm diameter, 29 cm height) filled with water (23 °C) to a height of 15 cm (to avoid climbing). The animals keep swimming until they give up, or they alternate between swimming and floating. A 6 min test session was video recorded and the last 4 min were scored (immobility time).

*Y-maze*: Working memory was assessed by recording spontaneous activity using a Y-maze, as reported [55]. The maze was made of gray acrilic plastic, and each arm was 25 cm long, 14 cm high, 5 cm wide, and positioned at equal angles. After each 8 min trial, the maze was cleaned with 70% ethanol to remove any olfactory cues. An off-line analysis of the videos was carried out to obtain the sequence of entries during the test. Alternate behavior was calculated as the percentage of real alternations (number of triplets with non-repeated entries) versus total alternation opportunities (total number of triplets).

*Social behavior*: We studied social novelty/preference in control mice, as described by others [53]. Each mouse was placed in a cage with three compartments (one central and two lateral arms, Figure S4D). In each compartment, and in an alternating manner to avoid possible compartment preference, we added a grid with one stranger mouse or an empty grid to assess social affiliation (intention to stay with the same species). We leave the mouse to explore for 10 min and record the time of direct interaction. Then, we cleaned the three chambers with ethanol and placed the mouse again in the center chamber. We include the previous stranger mouse in the same arm (now named “familiar mouse”). In the empty space we include a new mouse (“stranger mouse”) and leave the animal free to explore and recorded the time of direct interaction (Figure S4A,B).

### 4.4. Ca^++^ Fiber-Photometry

Fiber-photometry was used to assess in vivo neuronal activity through monitoring calcium dynamics, which serve as a proxy for neuronal firing. We stereotaxically injected an AAV- GCaMP virus (ssAAV-9/2-hSyn1-jGCaMP8m-WPRE-SV40p(A); Viral Vector Facility, ETH, Switzerland, 6.4 × 10^12^ vg/mL, 500 nL unilaterally injected, Figure S5) encoding a calcium-sensitive fluorescent indicator into the prefrontal cortex (AP = 1.7; ML = 0.5; DV = ±1.5) four weeks before the experiments, at a rate of 100 nl/min and the Hamilton syringe was withdrawn 10 min later. Mice were anesthetized with isoflurane (Zoetis, Parsippany, NJ, USA) administered with a nose mask (David Kopf Instruments, Paris, France), and placed on a stereotaxic frame (Stoelting Co., Wood Dale, IL, USA) on a heating pad and tape in their eyes to protect them from light.

*Peak Detection Analysis*: In the day of the experiment, mice received a bolus intraperitoneal (ip) injection of 1 µg/g body weight of IGF-1 dissolved in saline, as in previous work [56,57], or fasted for 20–24 h and ip injected with glucose (2 grs/kg). Mice were fasted for 20–24 h prior to glucose injection to maximize neuronal responses in fiber photometry, based on pilot studies indicating that shorter fasting yielded inconsistent signals. We acknowledge that this prolonged fasting deviates from standard recommendations and may represent an additional stressor. However, this prolonged fasting did not affect basal neuronal activity when compared with ad libitum fed mice. We use the R811 Dual Color Multichannel Fiber Photometry System (RWD Life Science, Shenzhen, China) to enable real-time detection and quantification of transient fluorescence signals (ΔF/F or Z-score) associated with neuronal activity in freely moving animal. In the preprocessing pipeline, 470 nm was selected as the signal wavelength, while 410 nm was designated as the control channel to correct movement artifacts and non-specific fluctuations. A smoothing filter (W coefficient = 15) was applied to reduce high-frequency noise without distorting signal integrity. To correct for fluorescence bleaching effects, baseline correction (PLS Fit algorithm, β coefficient = 8) was implemented, which is particularly suited for short and regular cycles of fluorescence decay. Furthermore, motion correction was applied to remove confounding artifacts caused by fiber displacement, ensuring that detected fluorescence variations represented genuine neuronal activity.

Following preprocessing, Peak Statistics parameters were configured for signal quantification. To refine the robustness of peak identification, the Median Absolute Deviation (MAD) Duration was set to 0.00–5.00 s, enabling precise calculation of the MAD value as a reference threshold. The Threshold for peak detection was adjusted to 1.00 MAD, ensuring that only fluorescence changes surpassing this criterion were considered significant. Additionally, Peak distances were set to 5.0 s, allowing for selecting the highest peak within each time frame while disregarding redundant peaks within the specified interval.

The Statistics Duration was set to 0–300 s at basal, defining the analysis window for peak detection, to assess neuronal activity changes, during which the peak count, frequency (Hz), and mean peak amplitude (% ΔF/F or Z-score) were quantified, with baseline activity considered the 100% reference. Subsequently, following the administration of IGF-1 or glucose, peak activity was evaluated within 5 min intervals, allowing for comparative analysis of neuronal responses to these interventions.

### 4.5. Immunoassays

Serum IGF-1 (Biotechne R&D Systems, Minneapolis, MN, USA), brain pSer^318^IRS-1/IRS-1 (Thermo-Fisher, Waltham, MA, USA), and serum corticosterone (Enzo Life Sciences, Redfern, Australia) levels were determined using commercial ELISAs, following the manufacturer’s instructions. Blood was collected at sacrifice at the same time of the day in all experimental groups to minimize circadian variations and obtain basal measurements. Sample selection was based on availability, as insufficient/hemolyzed serum volumes precluded analysis in some animals. The final cohort (1:1 male-to-female ratio) remained representative of all experimental groups.

We used immunocytochemistry to ascertain viral infection of VTA neurons. Animals were anesthetized with pentobarbital (50 mg/kg) and perfused transcardially with saline 0.9% and then with 4% paraformaldehyde in 0.1 M phosphate buffer, pH 7.4 (PB). Coronal 50-μm-thick brain sections were cut in a vibratome and collected in PB 0.1 N. Sections were incubated in permeabilization solution (PB 0.1N, Triton X-100, NHS 10%), followed by 48 h incubation at 4 °C with primary antibody in blocking solution (PB 0.1N, Triton X-100, NHS 10%). Antibodies used in this study include anti-NeuN (MAB377 Clone A60, Millipore Sigma, Burlington, MA, USA), and anti-GFP antibody (Rat IgG2a, monoclonal GF090R, Nacalai Tesque, Kyoto, Japan), followed by the respective polyclonal—Alexa Fluor (488/594) secondary antibodies (1:1000). Finally, a 1:1000 dilution in PB of Hoechst 33,342 was added for 5 min. Slices were rinsed several times in PB, mounted with gerbatol mounting medium, and allowed to dry. Images were taken with confocal microscopy (SP8, Leica Microsystems, Wetzlar, Germany).

### 4.6. Statistics

Statistical analyses were performed using GraphPad Prism 10 software (GraphPad Software, San Diego, CA, USA). Data distribution was first assessed with the Kolmogorov–Smirnov test for normality. Depending on the number of independent variables and the experimental design, group comparisons were conducted using one-way ANOVA followed by Tukey’s post hoc test or two-way ANOVA with Sidak’s multiple comparison test. In cases where repeated measures or hierarchical data structures were present, mixed-effects models (REML) were applied with appropriate corrections for multiple comparisons. For paired comparisons within the same subjects, paired t-tests were employed. When data did not meet parametric assumptions, the Kruskal–Wallis test followed by Dunn’s post hoc test was applied. For correlation analyses, simple linear regression was performed, reporting the regression equation, coefficient of determination (R^2^), slope with 95% confidence intervals, F statistics, degrees of freedom, and exact *p* values.

Sample sizes for each experiment were determined based on prior experience, aiming to achieve sufficient power to detect differences at a significance level of *p* < 0.05, while minimizing animal use in accordance with ethical standards. All animals were included in the analyses, and no exclusion criteria were applied a priori. Results are presented as mean ± standard error of the mean (SEM). Exact statistical values are reported in the Results and figure legends, including F/t values, degrees of freedom, R^2^, and exact *p* values, rather than significance thresholds.

## Data Availability

The original contributions presented in this study are included in the article/Supplementary Material. Further inquiries can be directed to the corresponding authors.

## Supplementary Materials

The following supporting information can be downloaded at: https://www.mdpi.com/article/10.3390/ijms262010008/s1.

## References

[1] Mattson M.P., Arumugam T.V. (2018). Hallmarks of Brain Aging: Adaptive and Pathological Modification by Metabolic States. Cell Metab..

[2] Fernandez A.M., Santi A., Torres Aleman I. (2018). Insulin Peptides as Mediators of the Impact of Life Style in Alzheimer’s disease. Brain Plast..

[3] Fernandez A.M., Torres-Aleman I. (2012). The many faces of insulin-like peptide signalling in the brain. Nat. Rev. Neurosci..

[4] Nishijima T., Piriz J., Duflot S., Fernandez A.M., Gaitan G., Gomez-Pinedo U., Verdugo J.M., Leroy F., Soya H., Nunez A. (2010). Neuronal activity drives localized blood-brain-barrier transport of serum insulin-like growth factor-I into the CNS. Neuron.

[5] Cheng C.M., Reinhardt R.R., Lee W.H., Joncas G., Patel S.C., Bondy C.A. (2000). Insulin-like growth factor 1 regulates developing brain glucose metabolism. Proc. Natl. Acad. Sci. USA.

[6] Bot M., Milaneschi Y., Penninx B.W., Drent M.L. (2016). Plasma insulin-like growth factor I levels are higher in depressive and anxiety disorders, but lower in antidepressant medication users. Psychoneuroendocrinology.

[7] Trejo J.L., Piriz J., Llorens-Martin M.V., Fernandez A.M., Bolos M., LeRoith D., Nunez A., Torres-Aleman I. (2007). Central actions of liver-derived insulin-like growth factor I underlying its pro-cognitive effects. Mol. Psychiatry.

[8] Zegarra-Valdivia J.A., Santi A., Fernandez de Sevilla M.E., Nunez A., Torres Aleman I. (2019). Serum Insulin-Like Growth Factor I Deficiency Associates to Alzheimer’s Disease Co-Morbidities. J. Alzheimers Dis..

[9] Santi A., Bot M., Aleman A., Penninx B.W.J.H., Aleman I.T. (2018). Circulating insulin-like growth factor I modulates mood and is a biomarker of vulnerability to stress: From mouse to man. Transl. Psychiatry.

[10] Mitschelen M., Yan H., Farley J.A., Warrington J.P., Han S., Herenu C.B., Csiszar A., Ungvari Z., Bailey-Downs L.C., Bass C.E. (2011). Long-term deficiency of circulating and hippocampal insulin-like growth factor I induces depressive behavior in adult mice: A potential model of geriatric depression. Neuroscience.

[11] Hawkes C.P., Grimberg A. (2015). Insulin-Like Growth Factor-I is a Marker for the Nutritional State. Pediatr. Endocrinol. Rev..

[12] Deuschle M., Blum W.F., Strasburger C.J., Schweiger U., Weber B., Korner A., Standhardt H., Gotthardt U., Schmider J., Pflaum C.D. (1997). Insulin-like growth factor-I (IGF-I) plasma concentrations are increased in depressed patients. Psychoneuroendocrinology.

[13] Jain S., Golde D.W., Bailey R., Geffner M.E. (1998). Insulin-like growth factor-I resistance. Endocr. Rev..

[14] Herrero-Labrador R., Trueba-Saiz A., Martinez-Rachadell L., Fernandez de Sevilla M.E., Zegarra-Valdivia J.A., Pignatelli J., Diaz-Pacheco S., Fernandez A.M., Torres Aleman I. (2020). Circulating Insulin-Like Growth Factor I is Involved in the Effect of High Fat Diet on Peripheral Amyloid β Clearance. Int. J. Mol. Sci..

[15] Pignatelli J., de Sevilla M.E.F., Sperber J., Horrillo D., Medina-Gomez G., Aleman I.T. (2022). Insulin-like Growth Factor I Couples Metabolism with Circadian Activity through Hypothalamic Orexin Neurons. Int. J. Mol. Sci..

[16] Simpson E.H., Akam T., Patriarchi T., Blanco-Pozo M., Burgeno L.M., Mohebi A., Cragg S.J., Walton M.E. (2024). Lights, fiber, action! A primer on in vivo fiber photometry. Neuron.

[17] Botterill J.J., Khlaifia A., Appings R., Wilkin J., Violi F., Premachandran H., Cruz-Sanchez A., Canella A.E., Patel A., Zaidi S.D. (2024). Dorsal peduncular cortex activity modulates affective behavior and fear extinction in mice. Neuropsychopharmacology.

[18] Wang Z.-J., Shwani T., Liu J., Zhong P., Yang F., Schatz K., Zhang F., Pralle A., Yan Z. (2022). Molecular and cellular mechanisms for differential effects of chronic social isolation stress in males and females. Mol. Psychiatry.

[19] Lowe C.J., Reichelt A.C., Hall P.A. (2019). The Prefrontal Cortex and Obesity: A Health Neuroscience Perspective. Trends Cogn. Sci..

[20] Hernandez-Garzon E., Fernandez A.M., Perez-Alvarez A., Genis L., Bascunana P., Fernandez de la Rosa R., Delgado M., Angel Pozo M., Moreno E., McCormick P.J. (2016). The insulin-like growth factor I receptor regulates glucose transport by astrocytes. Glia.

[21] Macauley S.L., Stanley M., Caesar E.E., Yamada S.A., Raichle M.E., Perez R., Mahan T.E., Sutphen C.L., Holtzman D.M. (2015). Hyperglycemia modulates extracellular amyloid-beta concentrations and neuronal activity in vivo. J. Clin. Investig..

[22] Trueba-Saiz A., Cavada C., Fernandez A.M., Leon T., Gonzalez D.A., Fortea O.J., Lleo A., Del S.T., Nunez A., Torres-Aleman I. (2013). Loss of serum IGF-I input to the brain as an early biomarker of disease onset in Alzheimer mice. Transl. Psychiatry.

[23] Zegarra-Valdivia J.A., Fernandes J., Fernandez de Sevilla M.E., Trueba-Saiz A., Pignatelli J., Suda K., Martinez-Rachadell L., Fernandez A.M., Esparza J., Vega M. (2022). Insulin-like growth factor I sensitization rejuvenates sleep patterns in old mice. Geroscience.

[24] Greene M.W., Sakaue H., Wang L., Alessi D.R., Roth R.A. (2003). Modulation of insulin-stimulated degradation of human insulin receptor substrate-1 by Serine 312 phosphorylation. J. Biol. Chem.

[25] Talbot K., Wang H.Y., Kazi H., Han L.Y., Bakshi K.P., Stucky A., Fuino R.L., Kawaguchi K.R., Samoyedny A.J., Wilson R.S. (2012). Demonstrated brain insulin resistance in Alzheimer’s disease patients is associated with IGF-1 resistance, IRS-1 dysregulation, and cognitive decline. J. Clin. Investig..

[26] Wilson S.J., Koffer R.E. (2025). Lonely days: Linking day-to-day loneliness to biological and functional aging. Health Psychol..

[27] Carro E., Trejo J.L., Busiguina S., Torres-Aleman I. (2001). Circulating insulin-like growth factor I mediates the protective effects of physical exercise against brain insults of different etiology and anatomy. J. Neurosci.

[28] Hendrickx H., McEwen B.S., van der Ouderaa F. (2005). Metabolism, mood and cognition in aging: The importance of lifestyle and dietary intervention. Neurobiol. Aging.

[29] Ong A.D., Uchino B.N., Wethington E. (2016). Loneliness and Health in Older Adults: A Mini-Review and Synthesis. Gerontology.

[30] Frystyk J., Brick D.J., Gerweck A.V., Utz A.L., Miller K.K. (2009). Bioactive insulin-like growth factor-I in obesity. J. Clin. Endocrinol. Metab..

[31] Nam S.Y., Lee E.J., Kim K.R., Cha B.S., Song Y.D., Lim S.K., Lee H.C., Huh K.B. (1997). Effect of obesity on total and free insulin-like growth factor (IGF)-1, and their relationship to IGF-binding protein (BP)-1, IGFBP-2, IGFBP-3, insulin, and growth hormone. Int. J. Obes. Relat. Metab. Disord..

[32] Lasselin J., Capuron L. (2014). Chronic low-grade inflammation in metabolic disorders: Relevance for behavioral symptoms. Neuroimmunomodulation.

[33] Ogrodnik M., Zhu Y., Langhi L.G.P., Tchkonia T., Krüger P., Fielder E., Victorelli S., Ruswhandi R.A., Giorgadze N., Pirtskhalava T. (2019). Obesity-Induced Cellular Senescence Drives Anxiety and Impairs Neurogenesis. Cell Metab..

[34] Atrooz F., Liu H., Salim S. (2019). Stress, psychiatric disorders, molecular targets, and more. Prog. Mol. Biol. Transl. Sci..

[35] Zegarra-Valdivia J.A., Pignatelli J., Nuñez A., Torres Aleman I. (2023). The Role of Insulin-like Growth Factor I in Mechanisms of Resilience and Vulnerability to Sporadic Alzheimer’s Disease. Int. J. Mol. Sci..

[36] Watanabe T., Miyazaki A., Katagiri T., Yamamoto H., Idei T., Iguchi T. (2005). Relationship between serum insulin-like growth factor-1 levels and Alzheimer’s disease and vascular dementia. J. Am. Geriatr. Soc..

[37] Adams M.M., Elizabeth Forbes M., Constance Linville M., Riddle D.R., Sonntag W.E., Brunso-Bechtold J.K. (2009). Stability of local brain levels of insulin-like growth factor-I in two well-characterized models of decreased plasma IGF-I. Growth Factors.

[38] Aguirre V., Werner E.D., Giraud J., Lee Y.H., Shoelson S.E., White M.F. (2002). Phosphorylation of Ser307 in insulin receptor substrate-1 blocks interactions with the insulin receptor and inhibits insulin action. J. Biol. Chem..

[39] Dietrich M.O., Muller A., Bolos M., Carro E., Perry M.L., Portela L.V., Souza D.O., Torres-Aleman I. (2007). Western Style Diet Impairs Entrance of Blood-Borne Insulin-like Growth Factor-1 into the Brain. Neuromol. Med..

[40] Kaiyala K.J., Prigeon R.L., Kahn S.E., Woods S.C., Schwartz M.W. (2000). Obesity induced by a high-fat diet is associated with reduced brain insulin transport in dogs. Diabetes.

[41] Carro E., Torres-Aleman I. (2006). Serum insulin-like growth factor I in brain function. Keio J. Med..

[42] Carro E., Torres-Aleman I. (2004). The role of insulin and insulin-like growth factor I in the molecular and cellular mechanisms underlying the pathology of Alzheimer’s disease. Eur. J. Pharmacol..

[43] Kappeler L., De Magalhaes Filho C.M., Dupont J., Leneuve P., Cervera P., Perin L., Loudes C., Blaise A., Klein R., Epelbaum J. (2008). Brain IGF-1 receptors control mammalian growth and lifespan through a neuroendocrine mechanism. PLoS Biol..

[44] Agrimi J., Spalletti C., Baroni C., Keceli G., Zhu G., Caragnano A., Matteucci M., Chelko S., Ramirez-Correa G.A., Bedja D. (2019). Obese mice exposed to psychosocial stress display cardiac and hippocampal dysfunction associated with local brain-derived neurotrophic factor depletion. EBioMedicine.

[45] Arnold S.E., Arvanitakis Z., Macauley-Rambach S.L., Koenig A.M., Wang H.Y., Ahima R.S., Craft S., Gandy S., Buettner C., Stoeckel L.E. (2018). Brain insulin resistance in type 2 diabetes and Alzheimer disease: Concepts and conundrums. Nat. Rev. Neurol..

[46] Kleinridders A., Ferris H.A., Cai W., Kahn C.R. (2014). Insulin action in brain regulates systemic metabolism and brain function. Diabetes.

[47] Kullmann S., Kleinridders A., Small D.M., Fritsche A., Haring H.U., Preissl H., Heni M. (2020). Central nervous pathways of insulin action in the control of metabolism and food intake. Lancet Diabetes Endocrinol..

[48] Kennard M.R., Nandi M., Chapple S., King A.J. (2022). The glucose tolerance test in mice: Sex, drugs and protocol. Diabetes Obes. Metab..

[49] Kim J., Kang H., Lee Y.B., Lee B., Lee D. (2023). A quantitative analysis of spontaneous alternation behaviors on a Y-maze reveals adverse effects of acute social isolation on spatial working memory. Sci. Rep..

[50] Chen Z., Sui G., Wang L., Yang C., Wang F. (2022). High-fat diet induced hippocampal CREB dysfunction, cognitive impairment and depression-like behaviors via downregulation of interleukin-2 in the mice. Metab. Brain Dis..

[51] Chen W., An D., Xu H., Cheng X., Wang S., Yu W., Yu D., Zhao D., Sun Y., Deng W. (2016). Effects of social isolation and re-socialization on cognition and ADAR1 (p110) expression in mice. PeerJ.

[52] Zhuang H., Yao X., Li H., Li Q., Yang C., Wang C., Xu D., Xiao Y., Gao Y., Gao J. (2022). Long-term high-fat diet consumption by mice throughout adulthood induces neurobehavioral alterations and hippocampal neuronal remodeling accompanied by augmented microglial lipid accumulation. Brain Behav. Immun..

[53] Huffman D.M., Farias Quipildor G., Mao K., Zhang X., Wan J., Apontes P., Cohen P., Barzilai N. (2016). Central insulin-like growth factor-1 (IGF-1) restores whole-body insulin action in a model of age-related insulin resistance and IGF-1 decline. Aging Cell.

[54] Munive V., Zegarra-Valdivia J.A., Herrero-Labrador R., Fernandez A.M., Aleman I.T. (2019). Loss of the interaction between estradiol and insulin-like growth factor I in brain endothelial cells associates to changes in mood homeostasis during peri-menopause in mice. Aging.

[55] Sarter M., Bodewitz G., Stephens D.N. (1988). Attenuation of scopolamine-induced impairment of spontaneous alteration behaviour by antagonist but not inverse agonist and agonist beta-carbolines. Psychopharmacology.

[56] Fernández de Sevilla M.E., Pignatelli J., Zegarra-Valdivia J.A., Mendez P., Nuñez A., Torres Alemán I. (2022). Insulin-like growth factor I mitigates post-traumatic stress by inhibiting AMP-kinase in orexin neurons. Mol. Psychiatry.

[57] Zegarra-Valdivia J.A., Pignatelli J., Fernandez de Sevilla M.E., Fernandez A.M., Munive V., Martinez-Rachadell L., Nunez A., Torres Aleman I. (2020). Insulin-like growth factor I modulates sleep through hypothalamic orexin neurons. FASEB J..

