# Supplementary material for: Influence of Lifestyle on Brain Sensitivity to Circulating Insulin-like Growth Factor 1
Source: Int J Mol Sci. 2025 Oct 14;26(20):10008. doi: 10.3390/ijms262010008 (PMC12562898; doi:10.3390/ijms262010008)
Supplement: Supplementary file 1 [file ijms-26-10008-s001.zip › ijms-3871292-supplementary.pdf]

Supplementary Information

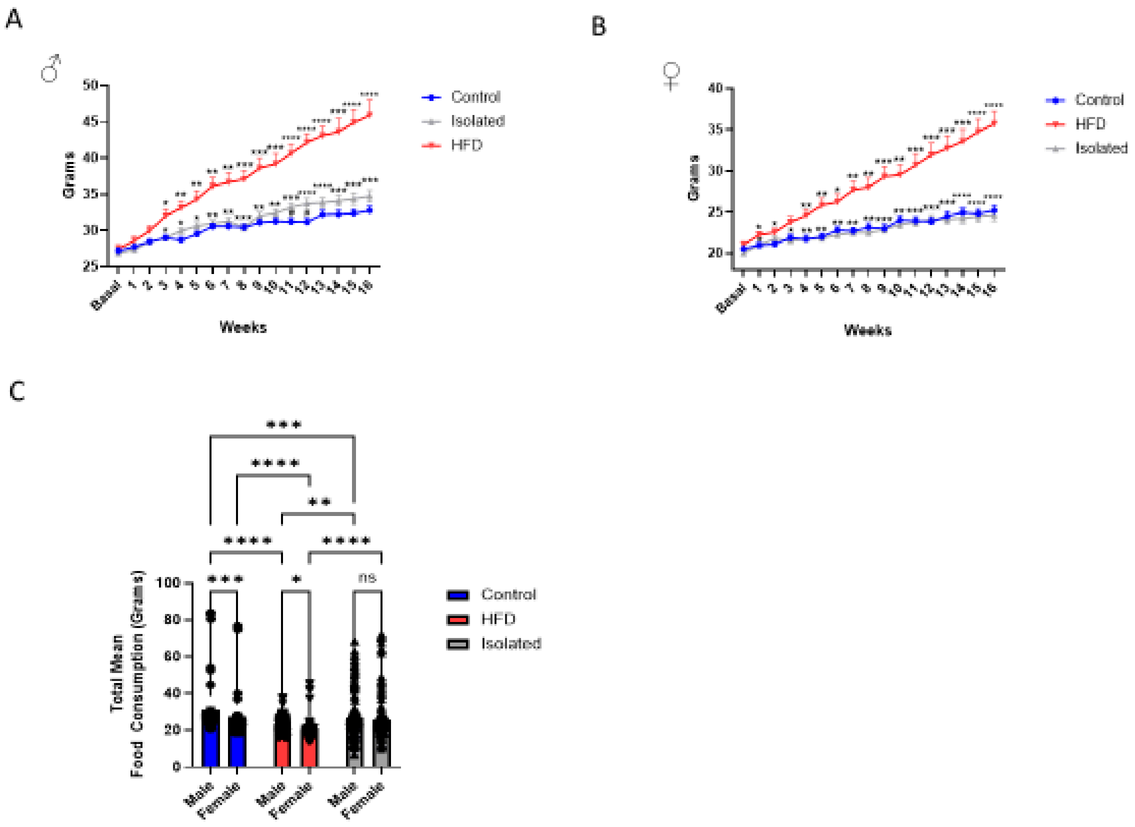

Supplementary Figure S1: A, B, Weight gain in male (A) and female (B) mice during the experimental period. HFD-fed mice of both sexes gained significantly more weight compared to controls. In males, isolated mice also showed statistical differences in weight compared to controls. In females, no significant differences were observed between isolated and control groups. Controls (males: n=12, females: n=13), HFD (males: n=13, females: n=13), Isolated (males: n=10, females: n=10). Results are presented as mean ± SEM. Two-way repeated measures ANOVA revealed a significant effect of time (males: $F(16, 76.92)=135.1$, $p<0.0001$; females: $F(16, 629)=35.79$, $p<0.0001$), dietary group (males: $F(2, 32)=23.45$, $p<0.0001$; females: $F(2, 629)=311.1$, $p<0.0001$), and their interaction (males: $F(32, 512)=21.36$, $p<0.0001$; females: $F(32, 629)=6.534$, $p<0.0001$). Post hoc Tukey's tests showed that HFD-fed mice had significantly higher weight than controls from week 4 onwards (males: week 4, $p=0.0156$; week 5, $p=0.0007$; week 7–16, all $p<0.0001$; females: all $p<0.0001$). In males, isolated mice also differed from controls at later time points (week 4: $p=0.0139$; week 5: $p=0.0002$; week 7–16: all $p<0.0001$). **C,** Sexual dimorphism in food

consumption was evident: males under standard or HFD diet ate more than females. Males, but not females, maintained on HFD or isolation consumed less than those on a normal diet (control group). Lower use of energy may explain higher weight gain in the face of reduced food consumption in isolated males. A two-way ANOVA revealed a significant effect of sex ($F(1,1140)=16.11$, $p<0.0001$) and diet ($F(2,1140)=40.63$, $p<0.0001$), but no sex × diet interaction ($F(2,1140)=1.894$, $p=0.1509$). Post hoc Tukey's tests showed that males consumed significantly more food than females (mean difference=4.31 g, 95% CI 1.93–6.69, $p=0.0004$). In males, both HFD and isolated groups ate less than controls (Control vs. HFD: mean difference=8.14 g, 95% CI 5.64–10.63, $p<0.0001$; Control vs. Isolated: mean difference=4.51 g, 95% CI 1.83–7.18, $p=0.0002$). In females, only HFD-fed animals showed reduced intake compared to controls (mean difference=6.17 g, 95% CI 3.32–9.01, $p<0.0001$). Male groups: Controls (n=10), HFD (n=13), Isolated (n=12). Female groups: Controls (n=13), HFD (n=12), Isolated (n=10).

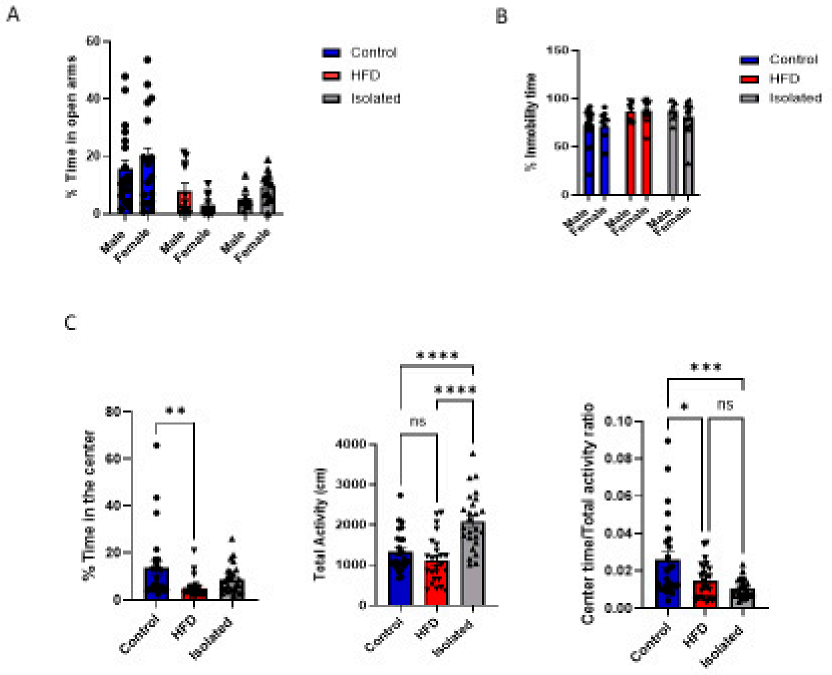

Supplementary Figure S2: A, No differences in performance were observed between males and females across groups in the Zero Maze test. Specifically, a two-way

ANOVA revealed no significant sex × diet interaction ($F(2, 76)=1.242$, $p=0.2947$) and no main effect of sex ($F(1, 76)=0.07869$, $p=0.7798$). In contrast, a main effect of diet was detected ($F(2, 76)=9.396$, $p=0.0002$). Post hoc Tukey's tests showed that in the control group males (n=20) and females (n=17) did not differ ($p=0.1773$), nor in the HFD group (males n=11, females n=11; $p=0.6608$) or the isolated group (males n=9, females n=14; $p=0.8419$). **B,** In the Forced Swim Test (FST), no sex differences were observed within any of the experimental groups. A two-way ANOVA revealed a significant effect of diet ($F(2,72)=7.934$, $p=0.0008$), but no effect of sex ($F(1,72)=0.8070$, $p=0.3720$), and no sex × diet interaction ($F(2,72)=0.3040$, $p=0.7388$). Post hoc Tukey's comparisons showed that within the control group, males (n=24) and females (n=15) did not differ significantly in immobility time (predicted LS means: 82.47 vs. 79.43 s, mean diff.=3.04, 95% CI [-3.71 to 9.80], $p=0.3720$). Similarly, in the HFD group, males (n=13) and females (n=12) displayed comparable immobility times (mean diff.=0.62, 95% CI [-6.29 to 7.53], $p=0.9812$). The isolated group also showed no sex-related differences (males n=9 vs. females n=14; mean diff.=2.11, 95% CI [-5.11 to 9.33], $p=0.6439$). **C,** Anxiety-like behavior was evaluated in the Open Field test (panel C). A one-way ANOVA revealed group effects on the percentage of time spent in the center ($F(2,71)=4.994$, $p=0.0093$). Post hoc analyses indicated that HFD-fed mice spent significantly less time in the center compared to controls, whereas isolated mice did not differ significantly from either group. Total locomotor activity also differed between groups ($F(2,72)=18.12$, $p<0.0001$), with isolated mice displaying hyperactivity compared to both controls and HFD-fed mice. To control for this increased activity, we calculated the ratio of center time to total activity, which also revealed significant group differences ($F(2,71)=7.868$, $p=0.0008$). Both HFD-fed and isolated mice showed a lower relative center exploration compared to controls, confirming heightened anxiety-like behavior.

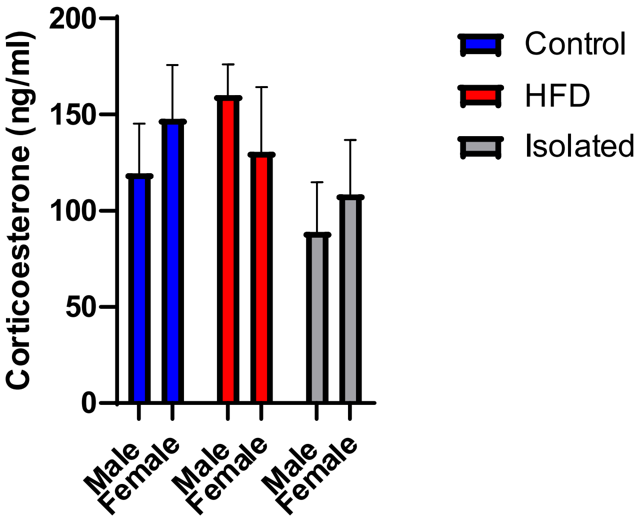

Supplementary Figure S3: Serum corticosterone levels in males and females across the three experimental groups (Control, HFD, and Isolated). A mixed-effects model (REML) was applied, revealing no significant effects of sex (F(1,8)=0.2490, p=0.6312), group (F(2,10)=1.378, p=0.2962), or sex × group interaction (F(2,8)=0.9430, p=0.4288). Pairwise post hoc comparisons with Šidák's test confirmed the absence of sex-related differences within any group (Control: mean diff. = –35033, 95% CI [–126097 to 56032], p=0.6284; HFD: mean diff. = 26768, 95% CI [–77391 to 130928], p=0.8445; Isolated: mean diff. = –19557, 95% CI [–119383 to 74869], p=0.9095). These results indicate that corticosterone levels did not differ significantly between males and females in any of the experimental conditions.

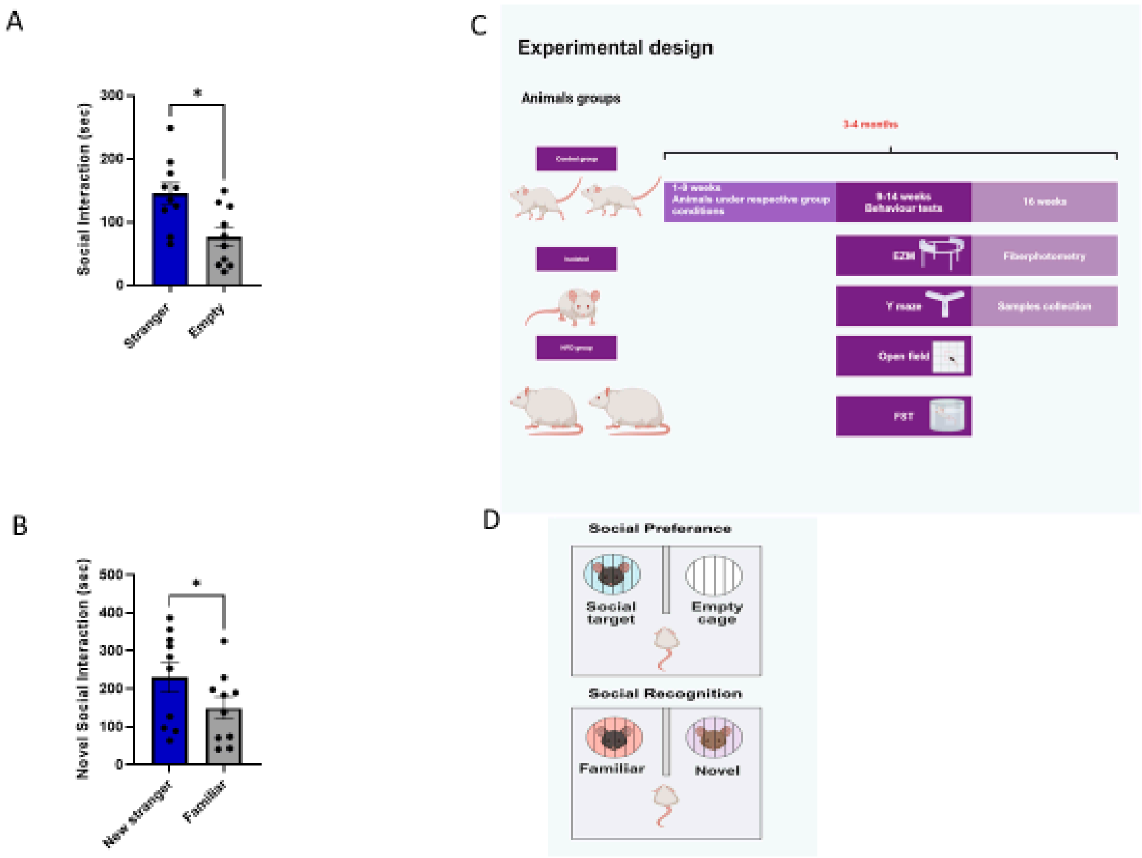

Supplementary Figure S4: A, Control mice spent significantly more time in the chamber containing a social partner compared to the empty chamber. A paired t-test confirmed this effect (t(9)=3.178, p=0.0112), with a mean difference of –68.39 seconds (95% CI [–117.1 to –19.71]). The effect size was moderate to large (R^2=0.5288), indicating robust sociability under control conditions (n=10).**B,** Control mice spent more time in the chamber containing a social partner than in the empty side. A paired t-test confirmed this effect, t(9)=3.178, p=0.0112, with a mean difference of –68.39 s (95% CI [–117.1 to –19.71]), indicating robust sociability in control animals (n=10). **C,** Experimental design: mice were divided into 3 groups (control, isolated and HFD) and kept during 9 weeks until behavioral assessments (week 14). Thereafter they were submitted to fiber-photometry or culled for sample collection. **D,** Scheme of the social interactions tests used.

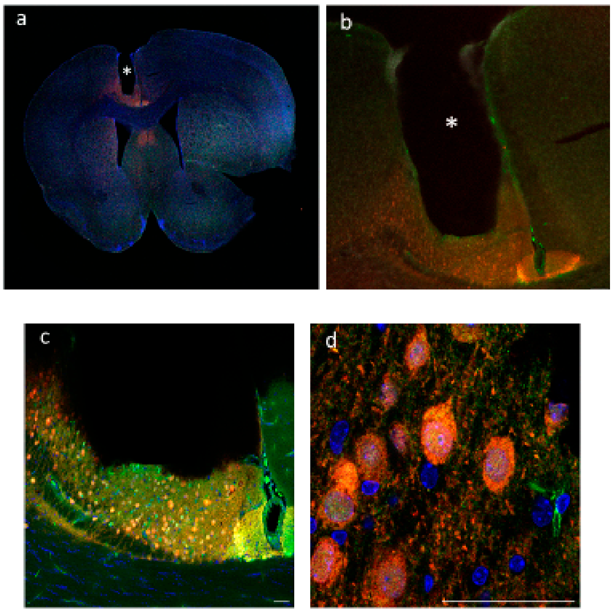

Supplementary Figure S5: Neurons (NeuN+ cells in green) in the PFC immunostained with GFP (red) after AAV- GCaMP virus injection. Note the trajectory of the fiber (*) on top of the prefrontal cortex area at lower magnification (a,b) . Double stained neurons are identified at higher magnification (c,d). Bars are 50 µm in (c) and 10 µm in (d). Cell nuclei are in blue.

Supplementary Table S1

Experiment (Figure/Panel)	Control (N)	HFD (N)	Isolated (N)	Notes
**Suppl. Fig. 1 – Weight gain / food intake**	Males: 10Females: 13	Males: 13Females: 12	Males: 12Females: 10	Separate by sex
**Suppl. Fig. 2A – Zero Maze**	M: 20 / F: 17	M: 11 / F: 11	M: 9 / F: 14	No sex differences, data pooled
**Suppl. Fig. 2B – Forced Swim Test**	M: 24 / F: 15	M: 13 / F: 12	M: 9 / F: 14	
**Suppl. Fig. 2C – Open Field (anxiety)**	37	22	23	Sex pooled
**Figure 1A – Zero Maze (main)**	37	22	23	Same as Suppl. Fig. 2C
**Figure 1B – Forced Swim Test (main)**	30	25	23	Slightly different N than Suppl.
**Figure 1C – Corticosterone**	9	7	8	ELISA
**Figure 1D – Y-maze**	19	25	25	Working memory
**Figure 2 – Fiber photometry (IGF-1)**	11	6	6	ΔF/F traces at baseline and 25 min
**Figure 3 – Fiber photometry (Glucose)**	9	9	7	Response at 15 min
**Figure 4A – pSer318-IRS1 (WB/ELISA)**	8	8	5	Cortical tissue
**Figure 4B – Serum IGF-1 (ELISA)**	9	10	10	Basal levels
**Figure 4C – Correlation serum IGF-1 vs. sociability**	10	–	–	Control group only

Distribution of animals across experimental groups and experimental techniques. Sample sizes correspond to each figure/panel as indicated.
